# Towards understanding the breast cancer epigenome: a comparison of genome-wide DNA methylation and gene expression data

**DOI:** 10.18632/oncotarget.6503

**Published:** 2015-12-08

**Authors:** Sandeep K. Singhal, Nawaid Usmani, Stefan Michiels, Otto Metzger-Filho, Kamal S. Saini, Olga Kovalchuk, Matthew Parliament

**Affiliations:** ^1^ Department of Oncology, Cross Cancer Institute, University of Alberta, Edmonton, Canada; ^2^ Service de Biostatistique et d'Epidémiologie, Gustave Roussy, Villejuif, France; ^3^ INSERM U1018, CESP, Université Paris-Sud, Villejuif, France; ^4^ Department of Medical Oncology, Dana-Farber Cancer Institute, Harvard Medical School, Boston, MA, USA; ^5^ Quantum Health Analytics SPRL, Liège, Belgium; ^6^ Department of Biological Sciences, University of Lethbridge, Lethbridge, Canada; ^7^ Canada Cancer and Aging Research Laboratories Ltd., Lethbridge, Canada

**Keywords:** DNA methylation, breast cancer, epigenetics, expression, microarray

## Abstract

Until recently, an elevated disease risk has been ascribed to a genetic predisposition, however, exciting progress over the past years has discovered alternate elements of inheritance that involve epigenetic regulation. Epigenetic changes are heritably stable alterations that include DNA methylation, histone modifications and RNA-mediated silencing. Aberrant DNA methylation is a common molecular basis for a number of important human diseases, including breast cancer. Changes in DNA methylation profoundly affect global gene expression patterns. What is emerging is a more dynamic and complex association between DNA methylation and gene expression than previously believed. Although many tools have already been developed for analyzing genome-wide gene expression data, tools for analyzing genome-wide DNA methylation have not yet reached the same level of refinement.

Here we provide an in-depth analysis of DNA methylation in parallel with gene expression data characteristics and describe the particularities of low-level and high-level analyses of DNA methylation data. Low-level analysis refers to pre-processing of methylation data (i.e. *normalization, transformation* and *filtering*), whereas high-level analysis is focused on illustrating the application of the widely used *class comparison*, *class prediction* and *class discovery* methods to DNA methylation data. Furthermore, we investigate the influence of DNA methylation on gene expression by measuring the correlation between the degree of CpG methylation and the level of expression and to explore the pattern of methylation as a function of the promoter region.

## INTRODUCTION

In order for a cell to function at the most basic level, its DNA encodes a core set of essential genes used to replicate, express and repair itself. These constitutive (“always on”) house-keeping genes are also responsible for controlling central metabolism. However, a milieu of other intricate cell-specific functions must also be maintained to ensure organismal functioning and survival. Furthermore, for an organism to develop, adapt and thrive, it must also interact and respond to its environment. In order to increase or decrease the production of specific reactionary gene products as needed, the vast array of genes are only activated at specific times and in specific tissue types. The process of switching genes “on” or “off” is known as gene regulation. Some core principles of gene regulation are preserved across all cellular organisms, albeit gene regulation processes are by far more complicated in eukaryotes compared to prokaryotes. In multi-cellular systems such as humans, cellular differentiation is determined by expression of different sets of genes allowing the incremental development of a diverse set of complex tissues and organs. The reason why a neuron, myocyte or hepatocyte exhibit different and distinct structural and functional characteristics is down to the differences in gene expression profiles. Similarly, a cancer cell acts differently from a normal cell for the same reason, i.e. the abnormal gene expression pattern.

Classically, an elevated disease risk has been ascribed to a genetic predisposition, however, exciting progress over the past years has discovered alternate elements of inheritance that mainly involve epigenetic regulation [1–2].

Characteristics that are propagated from cell to cell by some means other than changes in DNA sequence are referred to as epigenetic characteristics. Epigenetic changes are heritably stable alterations that include DNA methylation, histone modifications and RNA-mediated silencing [1]. Cytosine DNA methylation was the first epigenetic alteration identified, and it is the most widely studied epigenetic mechanism. It is crucial for the normal development, cell proliferation, as well as for the proper maintenance of genome stability in an organism [1, 3, 4, 5]. Altered methylation has been linked to the phenomena of global genomic instability and carcinogenesis [1, 3, 4, 6, 7]. DNA methylation is an addition of a methyl (CH_3_) group to the 5th carbon of the pyrimidine ring of cytosine resulting in a formation of 5-methyl-cytosine (5-me-C). S-adenosyl-L-methionine is a universal methyl-donor for this reaction which is catalyzed by DNA methyltranserases (DNMTs). Cytosine DNA methylation is the most common covalent base modification in the genome of vertebrates. In mammalian somatic cells, DNA methylation occurs predominantly at the cytosines within palindromic cytosine-phosphate-guanine (CpG) dinucleotides which tend to be methylated in a symmetrical fashion. In embryonic stem cells, though, DNA methylation occurs at both CpG and non-CpG sequences. DNA methylation is maintained through DNA replication by means of multi-protein complex containing DNMTs, methyl-CpG-binding, and histone-modifying proteins [3, 4].

Global genomic DNA methylation usually refers to the total overall content of 5-me-C in the genome. In mammalian genomes approximately 70–90% of CpGs are methylated, albeit CpG sites are not distributed evenly throughout the genome. CpG sites are located in intergenic DNA sequences, repetitive DNA sequences and exon other that the first exons. Short (< 4 kb) unmethylated genomic regions that contain high G + C content and high proportion of CpG dinucleotides are referred to as CpG islands. In normal cells, CpG islands are located at the 5′-ends of genes and in the intragenic and intergenic regions. Of the various types of CpG islands, those that span the promoter regions are mostly unmethylated [3, 4, 8]. The regulatory potential of DNA methylation manifests itself in the promoter regions that control the expression of adjacent genes. Hypermethylated promoters lead to an “off” state of expression, while those ones that are less methylated are deemed to lead to an “on” state [1]. Furthermore, methylated cytosines themselves can physically prevent the proper binding of transcription factors to promoter regions.

As such, DNA is crucial for normal development, cell proliferation, and maintenance of genome stability in an organism [9–11], and aberrant DNA methylation patterns are well-established characteristics of cancer cells [2, 5, 12, 13, 14]. Amongst those, DNA hypermethylation denotes the gain of methylation at particular sites that are unmethylated under normal conditions. Contrarily, DNA hypo-methylation constitutes the loss of DNA methylation at areas that are usually methylated [3, 4].

Global loss of DNA methylation has been associated with elevated mutation rates, activation of transposable elements, increased chromosome breakage, aneuploidy, and, thus with the phenomenon of global genomic instability [3, 4].

Importantly, novel approaches aimed to map DNA methylation across mammalian genomes uncovered reduced DNA methylation at regulatory regions but increased methylation in intergenic regions and repetitive sequences.

Recent advances in technology have made it possible to map DNA methylation patterns on a large scale (reviewed in Weber and Schubeler, 2007 [15]). A number of widely available commercial platforms exist that enable large-scale analysis of array-based DNA methylation. These include oligonucleotide or bead arrays (Illumina), lithographic arrays (Affymetrix), adaptive lithographic arrays (NimbleGen) and inkjet arrays (Agilent).

Bead array-based analysis of DNA methylation is one of the most commonly used techniques and is an extension of Illumina's genotyping method. In this technology, DNA is treated with bisulfite, which causes unmethylated cytosine on residues of the CpG dinucleotides to be converted into uracil while methylated cytosine remains unchanged [16].

Illumina has developed three array-based platforms named GoldenGate, Infinium 27 K and Infinium 450 K. The GoldenGate methylation profiling technology targets more than 1500 CpG sites throughout the genome, specifically those related to approximately 700 “cancer genes”. The Infinium methylation platforms, on the other hand, provide a broader “whole-genome” view. Infinium 27 K, utilizing the Infinium profiling technology on bisulfite-treated DNA, simultaneously assays the methylation status of more than 27,000 individual CpG sites, while the Infinium Human Methylation 450 K Bead Chip analyses more than 450,000 methylation sites. We accessed paired gene expression and methylation data, and used Infinium 27 K to describe some methods related to the analysis of DNA methylation patterns in human breast cancer.

**Table 1:** GEO information on the two series of data

	Reference	GEO number	Total	Normal	BC	Basal	Her2+	Luminal A	Luminal B	NA
**DNA Methylation**										
Series 1	Dedeurwaerder_et_al 2011	GSE20713	123	4	119	31	31	25	32	
Series 2	Dedeurwaerder_et_al 2011	GSE22249	125	8	117	35	25	27	30	
**Gene Expression**										
Series 1	Dedeurwaerder_et_al 2011	GSE20713	90	2	88	27	26	13	22	7

GEO: Gene expression omnibus

BC: breast cancer

NA: non-classified

Aberrant DNA methylation is a common molecular basis for a number of important human diseases, including breast cancer [17]. Breast cancer is a clinically and biologically heterogeneous disease. During the last decade, genome-wide gene expression microarray studies have made substantial progress and identified at least four different molecular subtypes of breast cancer with prognostic significance: basal-like, luminal A, luminal B and HER2+ [18–23]. The study of epigenetic changes in breast cancer may provide insight into the mechanisms of breast cancer progression and help develop tailored approaches for identification of risk factors, prevention, diagnosis and treatment. Indeed, CpG island hypermethylation is one of the most frequent mechanisms of loss of expression of a variety of critical genes related to breast cancer [24, 25]. There are many breast cancer-related genes (such as AKT, APAF1, APC, BCSG1, BRCA1, Caspase-8, CCND2, DAPK, E-Cad, ER, FHIT, GPC3, GSTP1, H_Cad, HIN1, HOXA5, NRF-2, p16, p21, p53, p73, PTEN, RASSF1A, RFC, SOCS1, SRBC, STAT1, SYK, THBS1, TIMP3, TMS1, ZAC, ZNF677) for which direct or indirect evidence suggests involvement of methylation [26–28]. These genes are involved in the regulation of cell proliferation, cell differentiation, programmed cell death, invasion, metastasis and immune recognition of tumor cells and other pathways [29, 30].

As more genome-wide DNA methylation data have become available, studies to unravel the intricate relationships between DNA methylation and gene expression have commenced. What is emerging is a more dynamic and complex association between DNA methylation and gene expression than previously believed. Although many tools have already been developed and refined for analyzing genome-wide gene expression data, tools for analyzing genome-wide DNA methylation have not yet reached the same level of refinement.

Based on the aforementioned, the main objectives of this article are therefore to provide an in-depth analysis of DNA methylation in comparison with gene expression data characteristics and describe the particularities of low-level and high-level analyses of DNA methylation data. Low-level analysis refers to pre-processing of methylation data (i.e. *normalization*, *transformation* and *filtering*), whereas high-level analysis is focused on illustrating the application of the widely used *class comparison, class prediction* and *class discovery* methods to DNA methylation data. Finally, we investigate the influence of DNA methylation on gene expression by measuring the correlation between the degree of CpG methylation and the level of expression and to explore the pattern of methylation as a function of the promoter region.

## RESULTS

### Comparison of DNA methylation and gene expression data characteristics

**Figure 1:**
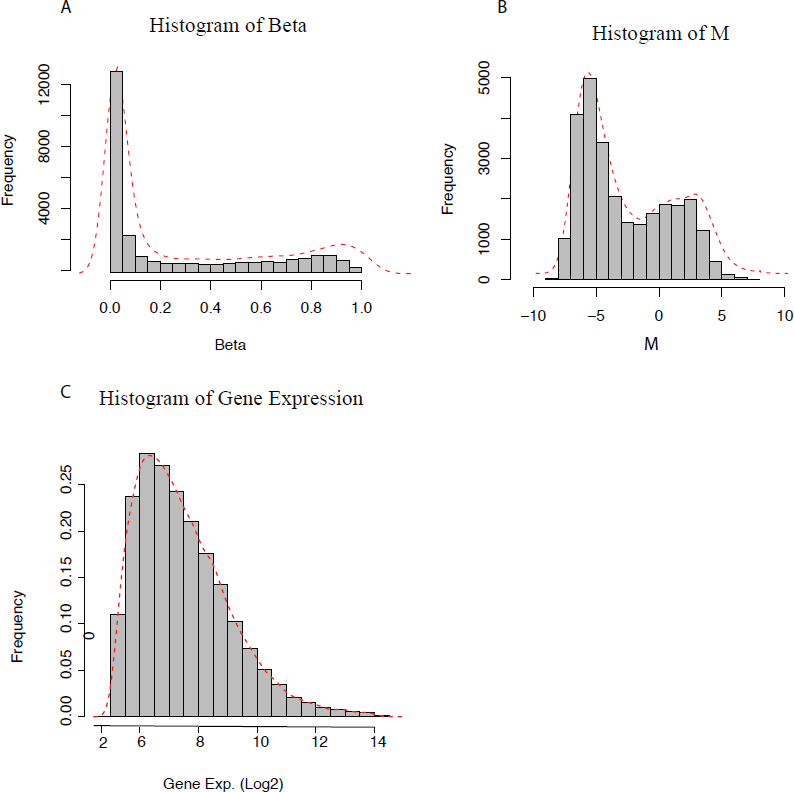
Distribution of DNA methylation and gene expression data (**A**) Methylation beta (β) values, (**B**) *M*-values, and (**C**) Log-transformed gene expression values.

We first compared data distribution by the density plot of DNA methylation and gene expression data. Figure 1A and 1C displays the density distribution of the DNA methylation β values across more than 27,000 CpG loci and gene expression log-normalized and transformed values for more than 54,000 probes for the same sample from Series 1 (Materials and Methods), respectively. The DNA methylation β value represents the absolute measurement for a given sample, and the distribution plot seems trimodal with a high peak of hypo-methylation and a low peak of hyper-methylation.

The global distribution of β values is consistent with the three reference categories [38] of the unmethylated standard showing low β values and having a large proportion of CpG sites characterized by low methylation, the hemi-methylated standard showing an intermediate β value and having a certain proportion of CpG sites in between the two peaks with moderate levels of methylation and the methylated standard having high β values and a smaller proportion of CpG sites characterized by high methylation. Thus, β values below 0.2, from 0.2 to 0.8 and above 0.8 [38, 39] were selected as threshold values to define unmethylated (including hypo-methylated), hemi-methylated and methylated (including hyper-methylated) CpG loci, respectively, for further analysis. When compared to gene expression data, DNA methylation data presents a skewed distribution, raising the question “Can we apply the same statistical methods for methylation data as applied for gene expression data?”

For most gene expression analysis, it is often assumed that the gene expression data are normally distributed after appropriate data normalization and parametric statistical tests, such as *t*-tests, analysis of variance (ANOVA) or linear regression are used [40], whereas, the observed DNA methylation distribution is trimodal and might lead to misleading results if the same test were to be applied. Presently, none of the normalization processes claim to convert β or M-distributions into the normally (or Gaussian) distributed form. Therefore, in the coming sections, we present some examples that show that nonparametric approaches are more suitable to analyze the methylation data.

**Figure 2:**
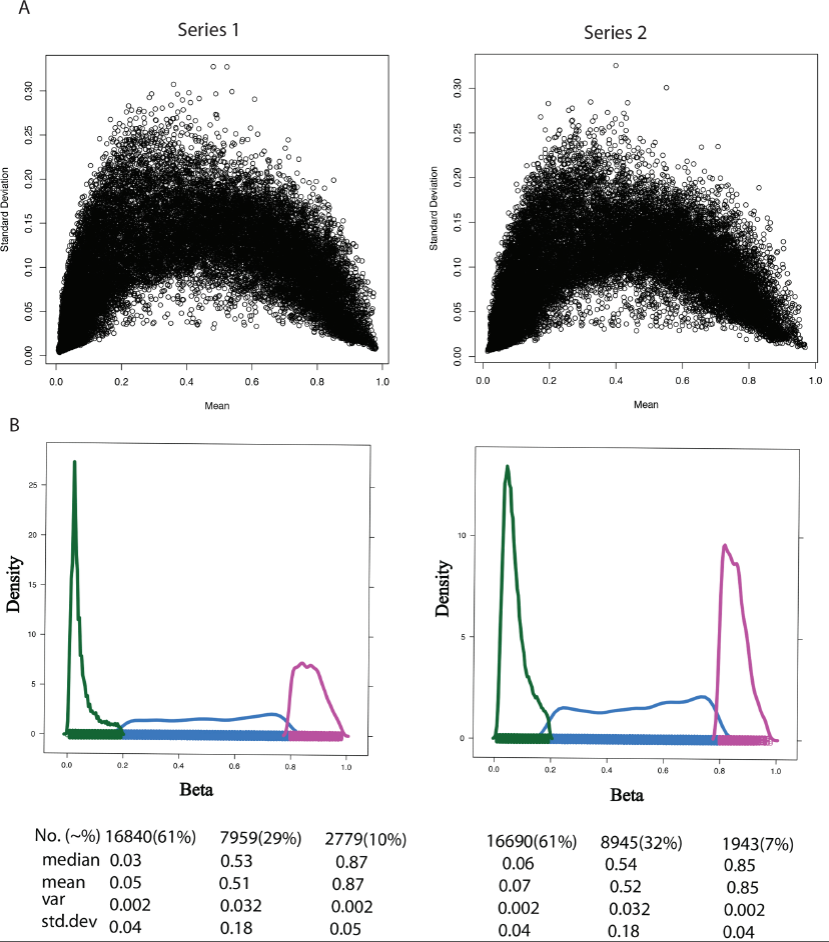
Variance measures with respect to mean β value across DNA methylation sample (**A**) The standard deviation (Y-Axis) and mean (X-axis) of β value for all samples in Series 1 (left) and Series 2 (right). (**B**) The properties of beta (β) value in the range of low [hypomethylated (UM), β-range 0 to 0.2, color green], medium [hemimethylated (HM), β-range 0.2 to 0.8, color blue], and high [hypermethylated (M), β-range 0.8 to 1.0, color purple] levels. Key statistical properties of the UM, HM, and M plots are highlighted below the figure. Table Abbreviations: No. represents Total number of CpGs in that range with percentage in bracket. Median, mean, var and std.dev represents median, mean, variation and standard deviation of CpG data in that range.

Next, we compared the variance distribution of DNA methylation and gene expression data. Figure 2A shows that the variance of the β values that have a mean closer to the centre of the range is much larger than the variance of the β measurement that has a mean closer to the limits of unmethylated or methylated values (i.e. β value 0 or 1). For example, if we divide the β value of a DNA methylation profile of one random sample from each of the two series according to three reference categories, i.e. unmethylated, hemi-methylated and methylated (Figure 2B), the variance of measurements with a mean for hemi-methylated is much larger (variance = 0.03) than the variance of measurements with a mean for unmethylated (variance = 0.0016) and methylated probes (variance = 0.002). In the case of gene expression, Archer et al. [41] show that the variance increases with increasing gene expression level.

To conclude, DNA methylation data do not have the same characteristics as gene expression data. Therefore, simply applying the statistical methods normally used for gene expression data to analyze DNA methylation data can introduce false positives to the results, unless such DNA methylation data are appropriately normalized and converted into a normal distribution. The next low-level analysis section will demonstrate some disadvantages if one applies the same methods.

### Low-level analysis

Data pre-processing is an essential step in the analysis of DNA methylation and gene expression microarray data. Principally, it consists of filtering the data using scanner information, background correction and normalization. The Bead Array technology from Illumina makes its pre-processing and quality control different from other microarray technologies. We will discuss some of these pre-processing steps for DNA methylation data below.

### Normalization of DNA methylation data using gene expression approaches

One basic assumption that is applied to the majority of the microarray gene expression data normalization methods is that most of the genes do not change across the biological samples being tested [42] and are referred to as housekeeping genes, which is not the case for DNA methylation data. A common way to illustrate this feature is by generating an MA plot which is a plot of log-intensity ratios (*M*-values) versus log-intensity averages (*A*-values) [43]. In most of the cancer studies, an MA plot provides information about how many genes are differentiated between two conditions such as cancerous versus normal cells.

In this example, we considered the average of the entire normal sample as reference for both gene expression and DNA methylation and drew the MA plot for the same sample of Series 1.

For gene expression, Supplementary Figure 3A shows the MA plot between reference and breast tumor sample. It demonstrates that the majority of the points on the *y*-axis were located at 0. If this is not the case, then one cannot apply normalization methods, such as quantile normalization and locally weighted scatter plot smoothing (LOWESS) normalization for microarray analysis. On the other hand, methylation patterns (or total amount of CpG methylation) can differ substantially among samples. Supplementary Figure 3B shows an MA plot between the mean of the reference and the same breast tumor sample using the β value. The Figure demonstrates that most of the points on the *y*-axis are away from the value 0. Therefore, normalization methods, such as quantile normalization and LOWESS normalization, may remove a true biological signal [40].

**Figure 3:**
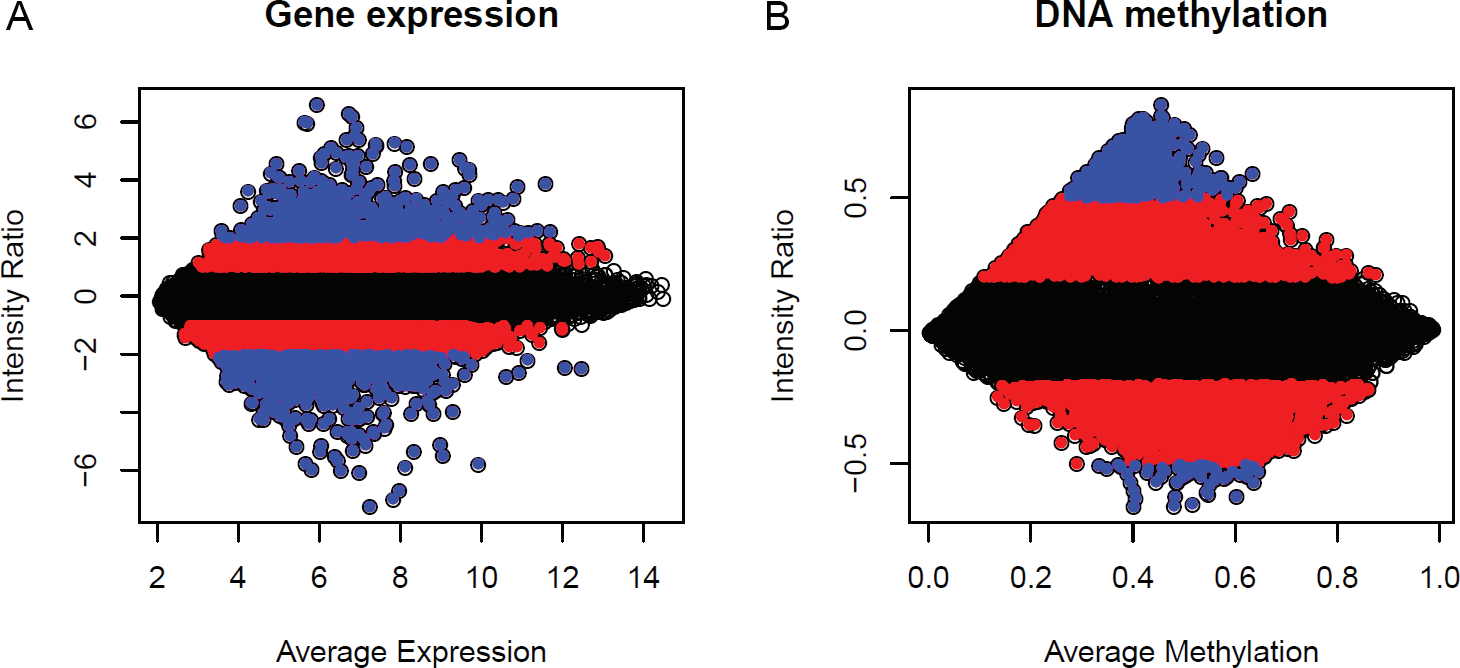
MA plots of normal averages (reference samples) versus a breast tumour sample M is, therefore, the intensity ratio, and A is the average intensity. Each dot represents one probe, or CpG, in the plot. (**A**) Gene expression; (**B**) DNA methylation. The three different colours represent the three ranges of intensity ratio gene expression ([−1, 1] black, [> 1, 2] and [< −1, −2] red, [> 2] and [< −2] blue and DNA methylation ([−0.2, 0.2] black, [> 0.2, 0.5] and [< −0.2, −0.5] red, [< 0.5] and [> −0.5] blue.

Another widely used assumption is that even if there are a large number of genes that are differentially expressed, there will often be an equal number of genes that are down-regulated and up-regulated [42]. The remaining genes are expected to have a constant expression and can therefore be used for normalization [44]. Similar assumptions cannot be made for DNA methylation measurements. First, we can see from Figure 3, the gene expression value in terms of a three different colour region shows approximately the same number of genes (dots) in the positive and negative directions, whereas the number of genes is different in DNA methylation, for example, the blue-colour region. Second, in general, methylation and unmethylation status are not independent. Recent genomic studies support the general inverse correlation between methylation and CpG density; higher CpG density is associated with lower methylation frequency [45–47], i.e. methylation status of a gene is mainly localized in the coding region, which is CpG poor. In contrast, the promoter region of the gene is unmethylated despite a high density of CpG islands in the region.

Supplementary Figure 1A shows the average of methylated and unmethylated signals across all samples of Series 1 with respect to distance from the transcription start site (TSS). In this Figure, the smoothed curve shows that the methylation signal increases as the curve moves away from the TSS. The opposite is observed for the unmethylated signal (Supplementary Figure 1B). The overall correlation between the methylated and unmethylated signal is shown in Supplementary Figure 2C.

According to the distribution of the methylation data and hypothesis used to normalize the gene expression data, the same procedures for DNA methylation data are not advisable. In this context, two different strategies can be applied to DNA methylation: 1) No normalization—use of raw average β values for analysis as recommended by Illumina [48]; 2) Quantile normalization followed by adjustment for batch, DNA input and bisulfite conversion efficiency effects [49].

There is thus a clear need for the development of more appropriate normalization methods for methylation data.

### Transformation of DNA methylation data into categorical form

The most common approach for transforming continuous DNA methylation data is to categorize the data into three categorical or discrete states according to unmethylated, hemimethylated and methylated β values. For example, Holm et al. transformed the data in a way that the continuous β values were reduced to three discreet values; β values ≤ 0.3 were set to 0, values > 0.3 and < 0.7 were set to 0.5 and values > 0.7 were set at 1 [50]. A similar approach with some modifications has been applied by Kamalakaran et al. [51, 52]. They used an expectation maximization algorithm with some modifications for estimating parameters for a mixture of three normal distributions.

In general, categorizing continuous data is not always beneficial since it can lead to a loss of statistical power [53]. Also, DNA methylation data do not have an underlying biology that dictates this discretization step as is the case for comparative genomic hybridization data where the copy number of discrete states reflects either a deletion, normal or amplification status [54]. For these reasons we do not encourage the discretization of continuous DNA methylation data.

However, a possible promising approach that needs further development for DNA methylation is to apply methods such as variance stabilization transformation, which may be used to transform the β value and stabilize the variance [40]. It is aimed at removing a mean and variance relationship so that the variance becomes constant relative to the mean.

### Filtering of DNA methylation data

Reducing the number of features by selecting probes with high variance or standard deviation or median absolute deviation is a common step in unsupervised as well as supervised analysis of gene expression microarray data.

The goal of filtering in methylation analysis is to select the specific CpG loci that meet certain condition(s). A frequent approach to reduce the number of genes (or probes) in the microarray is to select the most variant genes among all the samples (Appel and Ron 2009). However, this method could introduce a bias in the analysis of DNA methylation data. Indeed, as mentioned above, the variance is high for hemi-methylated CpG compared to unmethylated and methylated CpG loci; therefore, sorting features by variance or standard deviation may merely select hemi-methylated CpGs.

A second and preferred approach is to find the differentially methylated CpGs with respect to reference samples (for example, samples from normal mammary tissue in the context of the study of breast cancer tumors). In this case, we need to rank the CpGs using the difference between the methylation level of normal versus malignant tissue and then set a threshold *p* value to select all the CpGs lower than that value [17].

This second approach can further be refined by selecting the CpG having a significantly high/low methylation level compared to the normal sample in at least a certain number of tumor samples. For example, in Dedeurwaerder and Desmedt et al. [31], probes were selected having more than 20% methylation with respect to the mean of average βs for all normal samples in at least 30% of tumor samples. Therefore, the selected CpG loci indicate relatively high variability between normal and tumor samples.

### High-level analysis

In this section we will demonstrate some methods to perform a classification of patients by genetic profiling (DNA methylation data in this case), which is a crucial aspect of cancer prognostication and treatment. Most methods that we show at this point are already applied to gene expression data. We divided this section into three categories: class comparison, class prediction and class discovery. Class comparison refers to a powerful approach to identify the significantly differentially expressed or methylated loci (or genes) in different samples, which might be tissues, patients or cells exposed to different conditions. Class prediction is a supervised learning method similar to class comparison studies where the algorithm learns from samples with known class membership (training set) and establishes a prediction rule to classify new samples (test set). Such predictors can be used for many types of clinical management decisions, including risk assessment, diagnostic testing, prognostic stratification and treatment selection. In contrast, class discovery refers to discovering the clusters (subsets) of samples revealed by genetic profiles that are co-regulated or have similar behaviour or properties.

### Class comparison or subtype-specific epigenetic regulation

The specific objectives of this section are to determine whether the methylation profiles are different between breast cancer subtypes and, if so, to identify the differentially methylated loci. To provide a clinically and biologically relevant example, we performed a class comparison to identify the differently methylated CpGs among the four most common breast cancer subtypes (Basal-like, HER2+, Luminal A and Luminal B), using the nonparametric Kruskal–Wallis test. The β value was used to identify the subset of the most significant differently methylated CpGs. To overcome the problem due to multiple testing, the family-wise error rate (FWER) [55] approach was used.

In total, we found 99 CpGs (corresponding to 92 distinct genes) differently methylated between subtypes with FWER < 0.01 in Series 1 (119 breast cancer patients) and 571 CpGs (corresponding to 505 distinct genes) differently methylated between subtypes with FWER < 0.01 in Series 2 (117 breast cancer patients). Out of 99 CpGs of Series 1, we found 54 are common between Series 1 and Series 2. We used Series 1 as a training dataset and these 99 CpGs as key CpGs for further methods. The lists of differently methylated CpG loci in Series 1 and 2 are shown in Supplementary Table 1. On the basis of multiple corrected *p* values, results indicate strong differences in the methylation profile between the four molecular subtypes of breast cancer.

### Class prediction

Here, we aimed to demonstrate some DNA methylation-based predictor methods that accurately predicted the breast cancer subtype membership of a new sample on the basis of the methylation levels of key genes. A numbers of methods have been proposed that claim to successfully address this problem. Due to space limitations, we compare the performance of just two different approaches that can be applied to gene expression and DNA methylation data—nearest centroid classification (NCC) [56–58] and random forests (RF) [59–62]—through the comparison of misclassification (error) rates.

The NCC method depends on the assessment of similarity between objects. Dedeurwaerder and Desmedt *et al.* demonstrated this method could potentially identify a subset of CpG loci of methylation data that effectively discriminates subtypes of breast cancer [31]. Therefore, we examined the performance of the nearest centroid classifier coupled with a feature-selection algorithm. The process was done in two steps. First, using a training data set, we estimated the optimal nearest centroid classifier with a given number of features. Second, we compared the DNA methylation profile of a new sample to each of the class centroids determined using the training set. The predicted class of new sample was the one whose centroid was closest to the methylation profile of the test sample. A similar approach has been applied for breast cancer gene expression data analyses [20, 19].

In this example, we used Series 1 as a training data set and Series 2 as a test data set. With 99 key CpGs, we calculated four centroids (i.e. profiles consisting of the median methylation value for each of the 99 CpGs) for each of the four subtype-specific breast cancer groups (Supplementary Figure 2A). Since DNA methylation data are not normally distributed, we used a nonparametric distance measurement method (Spearman correlation) to measure the distance from centroids of the new sample. The Series 2 samples were then assigned to the nearest centroid (subtype) as determined by the highest Spearman correlation (Supplementary Figure 2B). Here, the misclassification rate in Series 2 was determined by calculating the number of samples differently classified as compared to the IHC status. The classification of all the samples of Series 2 resulted in a confusion matrix (Table 2A) that showed which samples were correctly classified (i.e. concordant with IHC status) and which were misclassified.

**Table 2:** Misclassification rate of class predication methods

Type	IHC groups	Grouping as per NCC output				
		Basal	HER2+	Luminal A	Luminal B	Error.rate
**A: Classification error rate: NCC centriod method**						
Basal	35	31	3	1	0	0.11
HER2+	25	3	15	3	4	0.4
Luminal A	27	1	2	21	3	0.22
Luminal B	30	5	3	13	9	0.7

Type	IHC groups	Grouping as per Random forest output					
	Total	Basal	HER2+	Luminal A	Luminal B	Error.rate
**B: Classificaon error rate: random forest method**						
Basal	35	30	4	1	0	0.14
HER2+	25	2	19	1	3	0.24
Luminal A	27	1	2	16	8	0.4
Luminal B	30	3	5	10	12	0.6

Column 1 represents the breast cancer subtypes by IHC and the rest of the columns represent the subtypes identified by centroid and random forest methods. The Error.rate column represents the difference in subtype classification according to IHC, and centroid and random forest methods, respectively.

The RF method proposed by Breiman et al. [63] is a combination of regression tree predictors such that each tree depends on the values of a random vector sampled independently and with the same distribution for all trees in the forest. It grows many classification trees and averages across the different trees. A similar approach has been applied to breast cancer gene expression data analyses Hu et al. [64].

This example was performed on DNA methylation average β values using randomForest R package version 4.5–34 [63]. RF values were generated by the key 99 key CpGs, which were then used to classify the Series 2 samples. Table 2B shows the confusion matrix of Series 2 samples using the RF method.

Both confusion matrices show similar misclassification results. The misclassification rate of Basal-like is minimum (11% and 14% with the NCC and RF methods, respectively), whereas Luminal A and B are the hardest to classify using DNA methylation data.

### Class discovery

To identify similar subgroups or partitions with methylation profiles, the two most commonly used clustering methods are hierarchical [52, 65] and recursively partitioned mixture modeling (RPMM) [66, 67].

Siegmund et al. evaluated a variety of methods for cluster analysis to determine the most reliable one [68]. They argued that the model-based approaches have lower misclassification rates compared to the heuristic hierarchical cluster analysis approach; however, this difference is less striking for discrete data than for continuous data. Houseman et al. showed that the model-based recursive-partitioning algorithm is more reliable than the competing nonparametric clustering approach for methylation data analysis [66]. Here, we demonstrate both approaches using Series 1 and 2 breast cancer DNA methylation data.

First, unsupervised hierarchical clustering was carried out by applying our reduced list of 99 key CpGs identified in Series 1 to the samples from Series 2. Subsequent β-values for these 99 key CpGs were clustered by using complete linkage and correlation distances with 1000 bootstrap replications by using an agglomerative clustering algorithm (pvclust), which is a Bioconductor package for hierarchical clustering with *p* values assessing cluster stability (developed by Suzuki and Shimodaira et al. [69]). Supplementary Figure 3A shows that two main clusters were formed, named cluster I and II. Cluster I was highly enriched with ER-positive samples (51/52 [98%]), whereas cluster II was enriched with ER-negative (46/63 [73%]) samples. Similarly, cluster II was more enriched with HER2+ samples (18/25 [72%]) compared to cluster I (7/25 [28%]). Since we did not find a complete separation of the four major breast cancer subtypes, we identified subtype-specific small clusters. Four subclusters (group1 in cluster I, group2, group3 and group4 in cluster II) were enriched with specific breast cancer molecular subtypes. Group1 was enriched with Luminal A (9/11 [82%]), group2 with Basal (24/29 [83%]), group3 with HER2 (8/11 [73%]) and group 4 with Basal-like (8/12 [75%]). The remaining samples did not show any subtype-specific clustering, but cluster I appeared to be enriched with Luminal (both A and B). On the other hand, the approximately unbiased (AU) *p* values were not that strong for the four clusters, and an AU *p* value less than 0.05 was used for the rejection of a given tree topology, as suggested by Suzuki et al. (Supplementary Figure 3B).

Second, we developed RPMM-based clustering for subgroup identification in β-distributed DNA methylation data, and demonstrated that this model outperformed the nonparametric (hierarchical clustering) methods in terms of classification error [66]. We applied RPMM to the methylation data of Series 2 with the 99 key CpGs.

**Figure 4:**
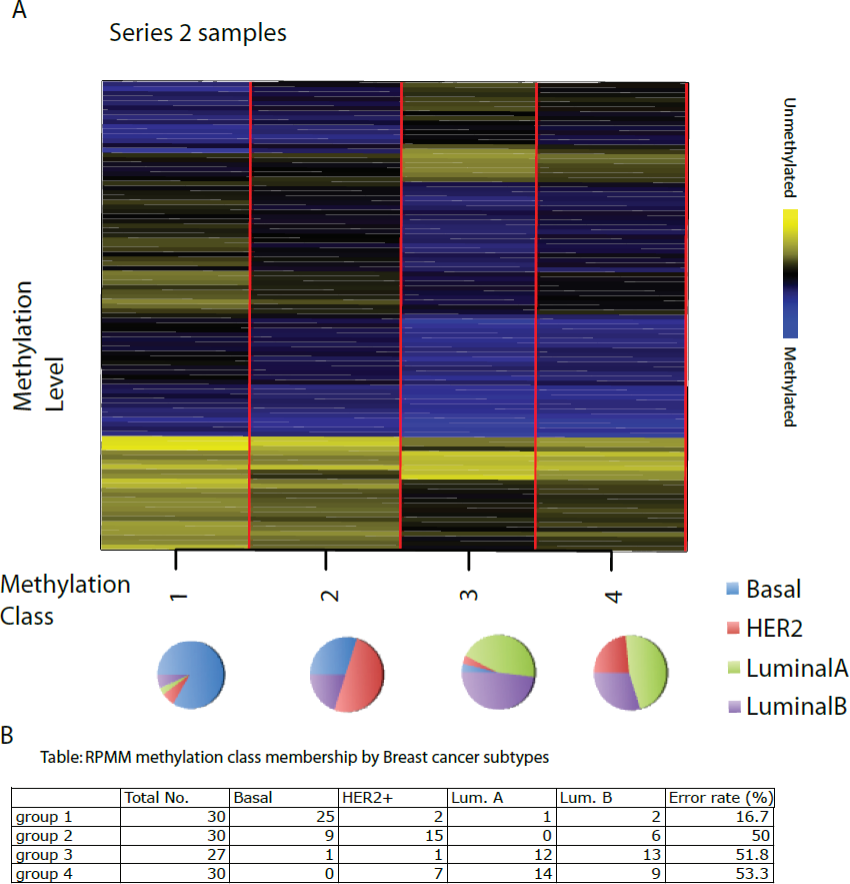
Recursively partitioned mixture model of CpG methylation in breast tumours The figure depicts the classification results of the RPMM analysis, trained on Series 1 and then applied to Series 2. The columns of heatmap represent CpG sites, and the rows represent methylation classes. The height of each row is proportional to the number of observations residing in the class (total *n* = 117). Color bar indicate blue as methylated and yellow as unmethylated level. The colour of the columns within each class represents the average methylation of the CpG for that class. The pie chart represents the proportion of different subtypes in each group. The classification results of Series 2 are shown in the Table below the Figure.

All 117 breast tissue samples resulted in four methylation classes (Figure 4). Separating samples into groups as Basal-like, HER2+, Luminal A and Luminal B, we found a significant association between group and methylation profile classes, with the majority of Basal-like (83%) residing in group 1, 50% of HER2+ in group 2 and most of the Luminals in groups 3 and 4. This also indicates that there is a relatively good separation based on the ER status, with groups 1 and 2 being enriched with ER-negative samples and groups 3 and 4 with ER-positive ones.

This comparative example shows that in terms of classification error, mixture models based upon the RPMM method perform better than the nonparametric clustering method.

### Integrated analysis of gene expression and methylation data

It is well know that DNA methylation is one of several epigenetic mechanisms that cells use to control gene expression. In order to better understand the impact of DNA methylation on gene expression, this last section shows some integrated analyses of DNA methylation and gene expression data.

### Correlation between methylation and expression data

A basic method to understand the relation between two or more variables is correlation analysis. Therefore, we investigated the nonparametric Spearman correlation between the DNA methylation and gene expression data of Series 1. The correlation was evaluated for 26,742 CpG loci and 13,871 gene expression probes (corresponding to 13,865 unique genes) identified by mapping (explained above) 88 breast tumors. The genome-wide correlation is shown in Supplementary Figure 4. The correlation ranges from −0.87 to 0.69. Inverse (rather than positive) correlations (167508/26473, i.e. 63.2%) between DNA methylation loci and expression probes were observed for many gene regulatory regions. We further analyzed the correlations within each breast cancer molecular subtype. We found that mean and median for all subtype-specific correlation was either 0 (in case of Luminal A) or shifted toward negative, indicating that more genes have inverse correlations between DNA methylation and gene expression than positive correlations. Complete statistical descriptions about the correlation results are shown in Supplementary Figure 4, and a complete correlation table is available in Supplementary Table 2.

We found a few subtype-specific anti-correlations. For example, *NAP1L5, MKRN3, CXCR3, SMOC1, CRYAB, VSIG9, LCP2, IL1R2, KLHL6* and *S100A4* were highly anti-correlated (correlation coefficient < −0.7) in Basal-like but not in the other subtypes. Similarly, an inverse correlation was found for *CASP10, CDKN1B, DAPK1, DAPK1, ESR1* and *TFE3* in the HER2+ subtype.

### Methylation and gene expression effects with respect to promoter/transcription start site

High-resolution methylation mapping allowed us to closely examine the relationship of DNA methylation to transcription. We mapped the DNA methylation data for each CpG to a promoter/TSS. We explored this region in detail, first using methylation data and then gene expression data. Using the Infinium platform, 27% of CpGs were found to lie in the region [−100, 100], whereas 78% CpGs were in the [−500, 500] region centred at the TSS. A histogram of the CpG corresponding to the TSS region is shown in Supplementary Figure 5.

A scatter plot was created to show the density distribution of the methylation level across the genome with respect to TSS by calculating the methylation level as an average across all the samples of the same series (Supplementary Figure 6, normal sample of Series 1 data has been used in this case). The red line shown in this figure is the smoothed curve (or the line of best fit) and describes the direction in which the points are heading. The figure shows that the methylation level is low in the region of [−500, 500] distance to the TSS and begins to increase as the distance from the TSS increases.

**Figure 5:**
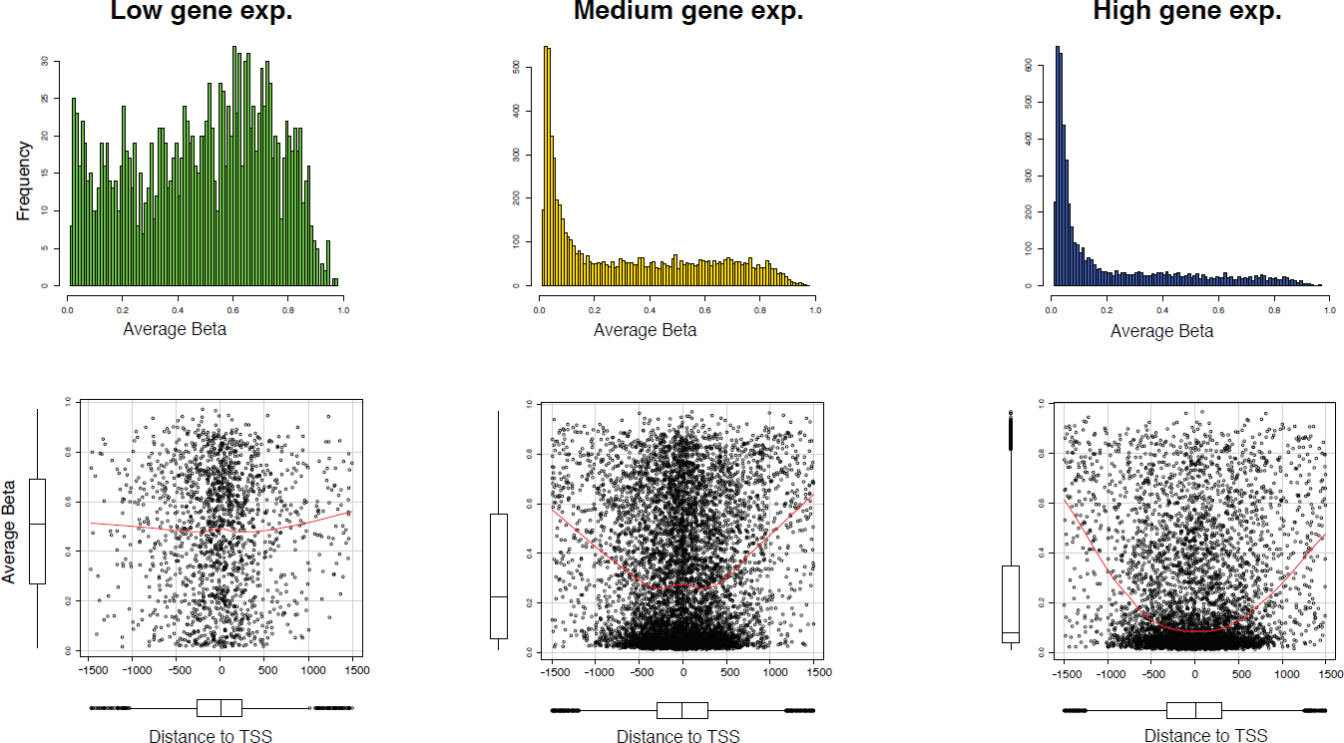
Association between DNA methylation and gene expression (**A**) Mean methylation levels according to three tertiles (low, medium, and high) of gene expression levels for 13,871 genes, identified by most variant mapping of Series 1 data. (**B**) Scatter plot of mean methylation levels in each gene expression tertile as a function of the distance from the transcription start site. The red line shows the smoothed curve. The distribution of average methylation values and data across the CpGs with respect to distance from transcription start site are visualized by boxplots.

To compare the expression and methylation levels in the entire genomic region, we calculated the average expression across all the samples of Series 1, and on the basis of the average gene expression, all probes were categorized into three classes, called low, medium, and high expression, for which the average expression of genes was less than the 1st quartile, between the 1st and 3rd quartile, and greater than the 3rd quartile, respectively. We then identified genes for which data were available both at the gene expression and DNA methylation level - 13,870 unique genes in this case. To dissect subtle methylation features that might be involved in the regulation of gene expression, we plotted the average methylation levels across the sample for the common genes corresponding to their gene expression classes. Figure 5 indicates that the genes with low expression have most of the hyper-methylated CpGs, whereas most of the highly expressed genes are hypo-methylated. Therefore, we can conclude that methylation levels and gene expression are, for the most part, inversely correlated within the promoter region.

## DISCUSSION AND CONCLUSION

The key goal of this study was to conduct an in-depth analysis of DNA methylation in conjunction with gene expression data characteristics and describe the particularities of low-level and high-level analyses of DNA methylation data. In this manuscript, we have endeavored to understand the key features of DNA methylation data and how they differ from gene expression data. Furthermore, we have proposed some computational approaches to analyze methylation data and compared them with the techniques available for gene expression analysis.

Data analysis begins with data preprocessing and quality control checks to detect and control for the effects of systematic technical errors while retaining biological variations. The pattern of data distribution should dictate which statistical approach is applied. The present study demonstrates that, in the case of DNA methylation array, the distribution curves of neither methylation β nor *M*-value are Gaussian. It also shows that methylation β distribution has a fixed scale ranging from 0 (un-methylated) to 1 (100% methylated) and, consequently, different statistical properties than gene expression arrays which are generally considered to have an infinite scale. It is important to note that the mean distribution of variance across the samples is not uniform, so applying feature selection methods based upon variations could produce biased results. Generally, gene expression microarray data are converted into a normally distributed form before they are applied to gene expression microarray data. However, most techniques for such normalization require two basic conditions: 1) the presence of housekeeping genes; and 2) an approximately equal number of up- and down-regulated genes. These conditions do not hold true for methylation β or *M*-value data; therefore, such normalization techniques are not applicable when normalizing methylation data. In addition, transforming continuous DNA methylation data into discrete categories leads to a loss of statistical power. This indicates a clear gap in research on pre-processing of methylation array data and the need for future development and refinement of appropriate solutions.

In a high-level analysis, we illustrate the statistical approaches that can be used to achieve the specific goals of different DNA methylation studies (class comparison, prediction and discovery) and to elucidate the relationships between DNA methylation data and gene expression. The objective of class comparison methods is to identify the distinguishing features of the classes. Due to the non-normal distribution of DNA methylation data, a nonparametric approach seems to better suit the properties of the data necessary to identify the different methylated features. In the context of class prediction, researchers are generally interested in developing a model that can correctly assign individual patients to the appropriate categories. A number of tools can be utilized for supervised classification with high-dimensional genomics data.

We show the application of two frequently used approaches (NCC and RF) to gene expression array data. The two approaches have little differences in their misclassification rate, or the proportion of cases classified incorrectly. In class discovery, the classes are not known in advance, and different classes can be obtained from the same dataset by selecting different methods or parameters. The aim is to exploit the redundancy in the data and to identify the subset(s) of data that share certain features. In this study, we explore two methods: (1) hierarchical clustering, which is a popular method used with microarray data with unknown number of classes; and (2) RPMM, which employs a recursive-partitioning algorithm to navigate clusters in a beta mixture model that provides the number of clusters. As claimed in this article, RPMM provides a reliable solution in less time than sequential attempts with different numbers of assumed clusters. The results are then compared with the breast cancer sub-classifications identified by IHC. The output of both methods is compared with the classification error rate, or the number of samples in such clustering classes with respect to IHC classification. This study demonstrates that the application of RPMM results in a low error rate.

The section of genome-wide comparative analysis of DNA methylation and gene expression with respect to TSS shows that DNA methylation in the range from TSS [−500, 500] bp (which can be a promoter) is strongly anti-correlated with gene expression and low levels of anti–correlation beyond this region.

As with other topics in the field of human epigenetics, gaps remain in our knowledge of DNA methylation. To deal with these gaps, a systems approach is needed that includes (1) data collection and integration of all available information, (2) low level data analysis that includes the development of adequate computational methods/tools for data cleaning and preprocessing to remove noise and outliers/wild shots, and handling of missing data; (2) transforming data to find useful features which represent data more efficiently; (3) high level system modeling with a good algorithm that has a good predictive power; and (4) generation of new hypotheses to explain the positive correlation between gene expression and DNA methylation.

In conclusion, the nonparametric methods presented in this overview seem more appropriate to the analysis of DNA methylation data and can be straightforwardly applied to other studies in order to pin down novel cancer genes whose expression is altered by DNA methylation alone

In sum, our analysis is consistent with the previous notion that DNA methylation patterns occurs at well-defined regions and are associated with aberrant gene expression patterns [15]. Similarly, our analysis also suggests that hyper-methylation may in turn be the default state of human genome and that dynamic DNA methylation changes occur during carcinogenesis and are associated with cancer. In the future it would be important to conduct an in-depth analysis of entire milieu of signalling pathways in breast cancer taking into consideration genetic as well as epigenetic phenomena that impact the breast cancer signalome [70–72].

Moreover, the most recent genome mapping experiments have identified the presence of 5-hydroxymethylcytosine (5-hm-C) in the genome and provided the initial insight into the role of 5-hm-C in metabolism of 5-m-C and active DNA demethylation. Its effects on gene expression have yet to be fully established [73–78]. Therefore, in order to get a full understanding of (epi)-genome functioning in normal and cancer cells, in the future it would be important to determine the inter-relationship between DNA methylation, DNA hydroxymethylation and gene expression, and their regulation in normal and cancer cells and tissues.

## MATERIALS AND METHODS

### Data sets

DNA methylation and gene expression data were obtained from the publicly available Gene Expression Omnibus database (http://www.ncbi.nlm.nih.gov/geo/, GSE20713, GSE22249). The studies used in this paper are shown in Table 1. Series 1 and 2 were published in the same article, but Series 2 has no gene expression data available. The breast cancer subtypes basal, HER2+, luminalA and luminalB were classified according to ER and HER2 status by immunohistochemistry (IHC) and histological grade as described by Dedeurwaerder and Desmedt et al. [31].

### Methylation data

We used a breast cancer data set consisting of 248 samples, subdivided into Series 1 (total =123, includes 119 breast and 4 normal) and Series 2 (total =125, includes 117 breast and 8 normal) as described previously [31]. Methylation analysis was performed using Infinium Methylation 27 K arrays (Illumina, San Diego, CA) [32]. This array generates data on a large number of informative loci (27,578 CpG sites from 14,495 protein-coding gene promoters and 110 microRNA gene promoters) for each sample at single-site resolution. GenomeStudio^™^Methylation Module v1.0 was used for data extraction and quality control.

### Gene expression data sets

Affymetrix expression data for 90 out of 123 samples of Series 1 has been described previously (Dedeurwaerder and Desmedt 2011). Expression analysis was performed using the HG U133 Plus 2.0 chips from Affymetrix (54675 probe sets). The data were analyzed by using the quantile normalization method of the “affy” package [33] available from http://www.bioconductor.org/. No background correction was performed for the analysis in this paper. Subsequently, data were log2 transformed. As mentioned above, no gene expression data were available for Series 2.

### Mapping between gene expression and DNA methylation

Hybridization probes were mapped to Entrez GeneID as described [34] using RefSeq and Entrez database version 2007.01.21. Two types of mapping have been used for the analysis: maximum (many-to-one) mapping and most variant (one-to-one) mapping.

For maximum mapping, all CpG loci of methylation data were mapped on the basis of Entrez Gene ID to the available gene expression data. Using this method we identified 26,472 CpG corresponding to 13,871 gene expression probes of Series 1 data of Dedeurwaerder and Desmedt (2011) Supplementary Table 3A.

For most variant mapping, all CpG loci of methylation data were mapped on the basis of Entrez Gene ID to the available gene expression data; the one with the highest variance in expression data set was selected. There were 13,871 genes in common between methylation and expression data of Series 1 of Dedeurwaerder and Desmedt et al. Supplementary Table 3B.

### Methylation level measurement

The methylation level was analyzed using a paired (methylated and unmethylated) probe set. To date, two methods have been proposed to measure the methylation level: the beta value (β) and the *M* value [35].

The β value has been widely used to measure the percentage of methylation. In this method, the methylation status is measured as a fraction of fluorescence signal from methylated molecules over the sum of fluorescence of methylated (Me) and unmethylated (Um) molecules, i.e. β = (Me)/(Me + Um + 100). Raw average β values were analyzed without normalization, as recommended by Illumina. The data range was a continuous value between 0 (i.e. unmethylated) and 1 (i.e. 100% methylated). This is the method currently recommended by Illumina [36, 37].

The *M* value refers to the log2 ratio of the signal intensities of the methylated probe versus the unmethylated probe. The comparison between the β value versus the *M* value has been described by Du et al. [35], who claim that the *M* value is statistically more robust for the differential analysis of methylation levels. Figure 1A and 1B shows the distribution of β and *M* value, respectively, for the same sample of the Series 1 data set.

## SUPPLEMENTARY FIGURES AND TABLES










