# Supplementary material for: Towards understanding the breast cancer epigenome: a comparison of genome-wide DNA methylation and gene expression data
Source: Oncotarget. 2015 Dec 8;7(3):3002–17. doi: 10.18632/oncotarget.6503 (PMC4823086; doi:10.18632/oncotarget.6503)
Supplement: Supplementary file 1 [file oncotarget-07-3002-s001.pdf]

Towards understanding the breast cancer epigenome: a comparison of genome-wide DNA methylation and gene expression data

Supplementary Materials

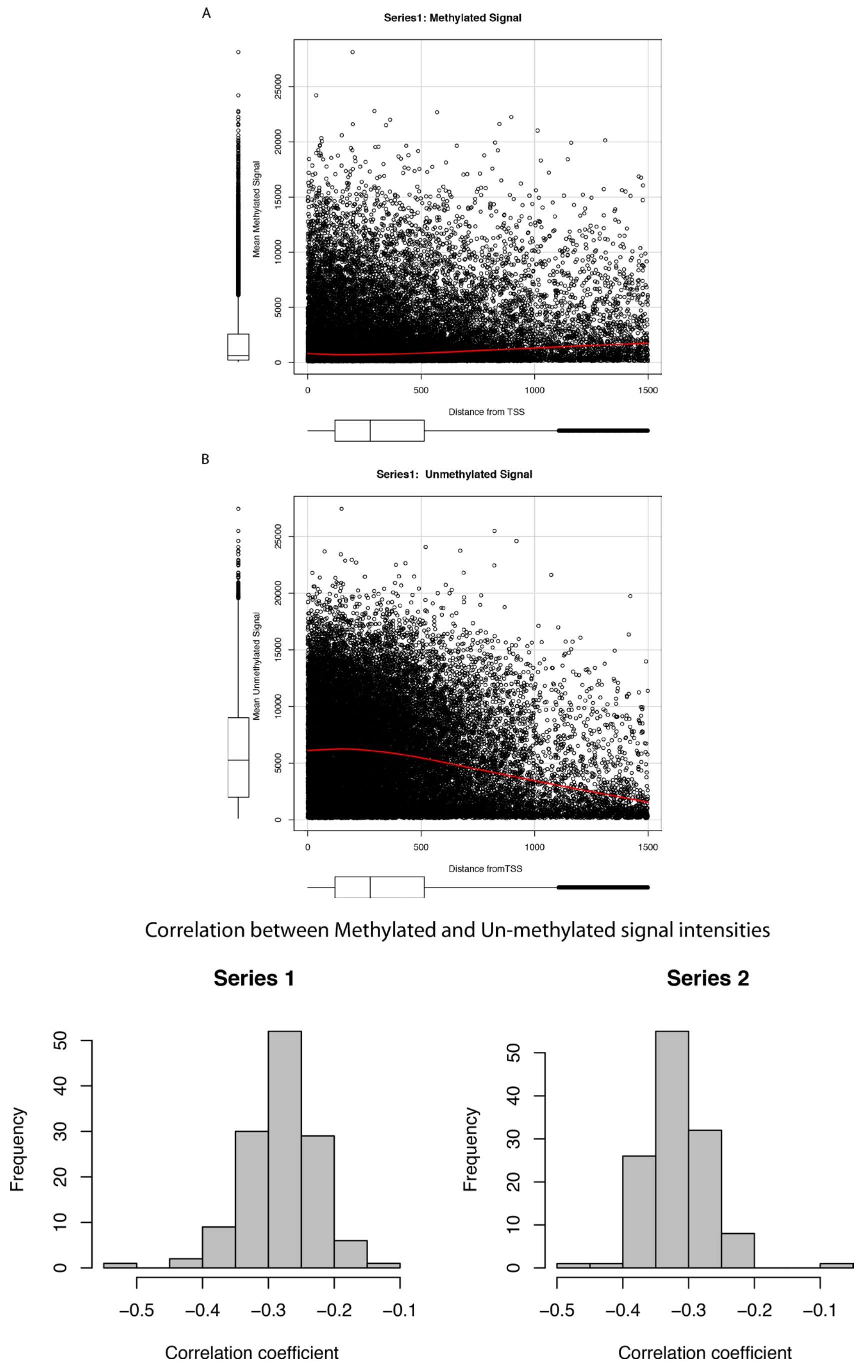

Supplementary Figure S1: Scatter plot of the (A) average raw methylated and (B) average raw unmethylated signal intensities across all samples of Series 1 data as a function of distance from the transcription start site. The red line shows the smoothed curve. The distribution of the signal intensities and distances are visualised by boxplots. (**C**) A histogram of correlation values between average methylated and unmethylated raw signal intensities of CpG loci across all of the samples from Series 1 and Series 2.

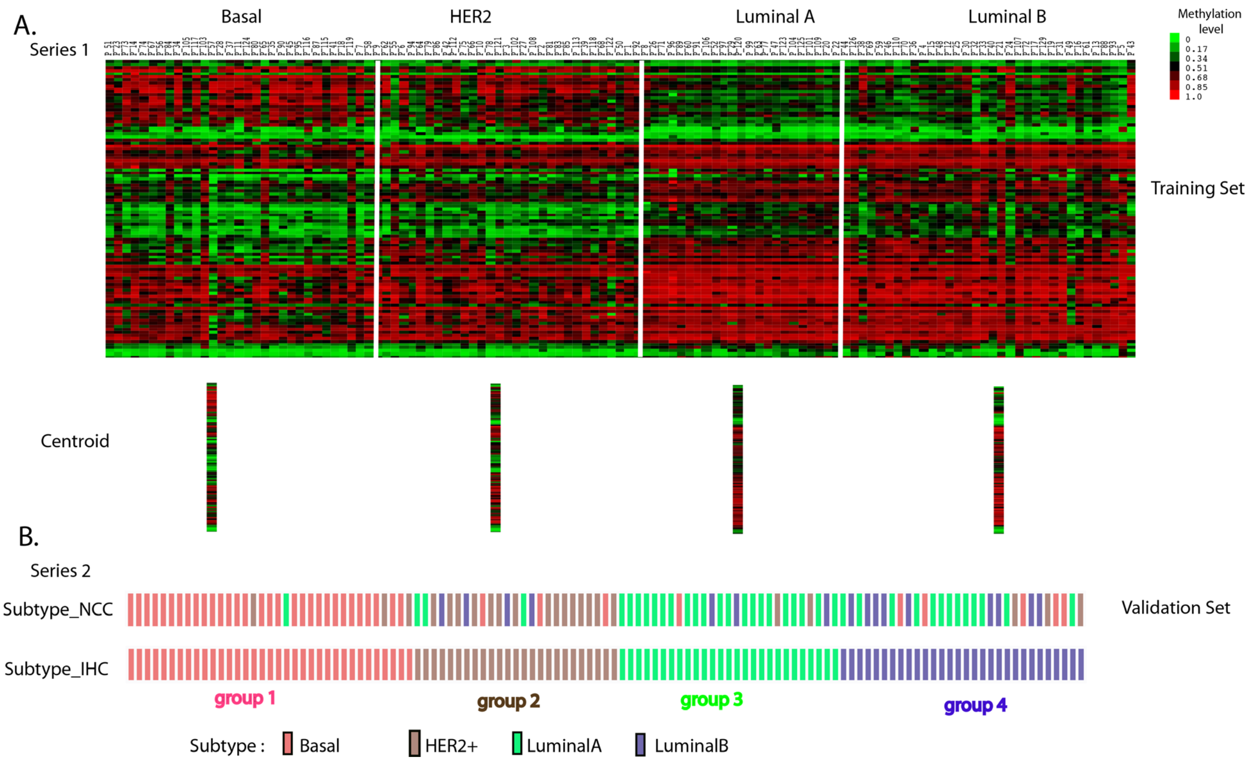

Supplementary Figure S2: Nearest centroid classification (NCC) model of CpG methylation in breast tumours. (**A**) Heat map of the centroid models of the subtype using 99 CpGs and 119 samples of Series 1 as a training set. The centroids were constructed by calculating the median of 99 CpGs within each subtype. The beta (β) values are shown as red, black or green according to their relative methylation levels. (**B**) The figure depicts the results of NCC on the samples of Series 2 data (117 BC samples) used as a validation set. The first row represents the predictive breast cancer classification of samples according to the NCC method (Subtype_NCC). The second row represents the samples classified by immunohistochemistry (IHC) results, the colours being green (luminal A), purple (luminal B), brown (HER2+), and pink (ER−/HER2−).

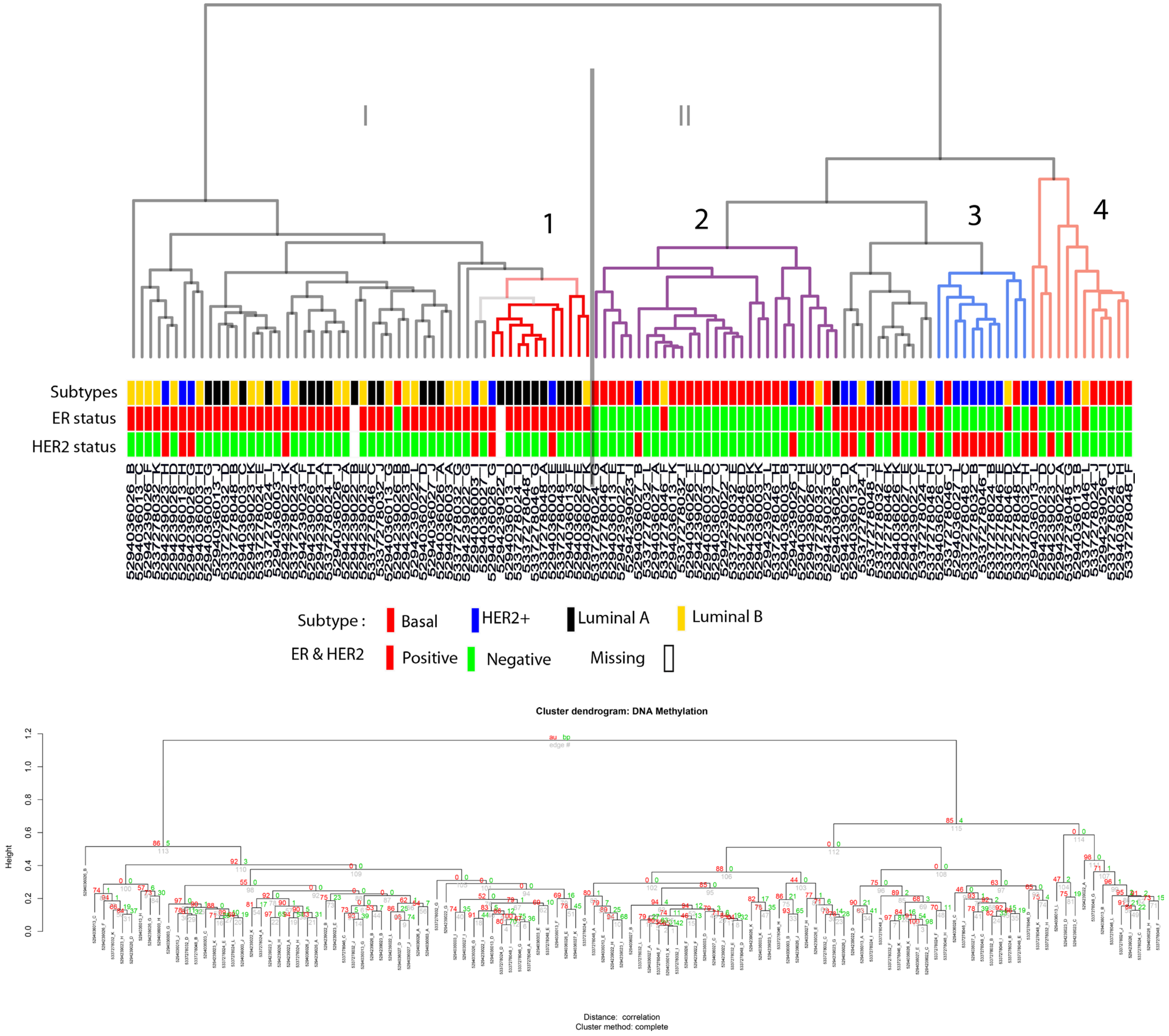

Supplementary Figure S3: (**A**) Hierarchical clustering of CpG methylation on breast tumour samples from Series 2 ($n = 117$). Clustering was performed on the 99 key CpGs identified as being differently methylated among four breast cancer subtypes with distance correlation and complete linkage. Below the dendrogram, the coloured table represents the subtype characteristics of corresponding samples. (**B**) The approximately unbiased (AU) (red) and bootstrap probability (BP) (green) values of hierarchical clustering were calculated by the pvclust method. Figure 3A, clustering of the validation dataset shows two main clusters (I and II). Cluster I was enriched with ER+/HER2−. Additionally, four subclusters were selected with enrichment of one particular subtype, such as subcluster I was enriched with luminal A (black), subclusters II and IV were enriched with basal (red), and subcluster III was enriched with HER2+ (blue) samples.

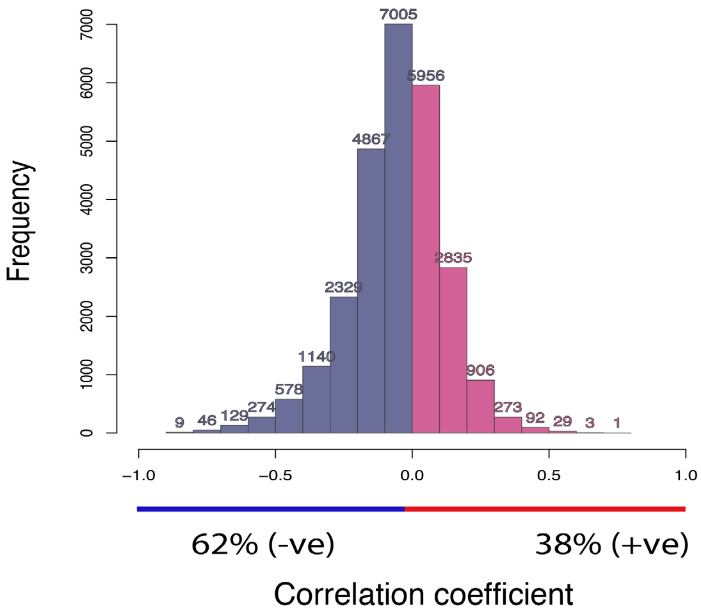

Correlation statistics

Series 1	No. sample	% Negative Corr.	Max. Negative Corr.	Max. Positive Corr.	Mean Corr	Median Corr.
Total	88	16508/ 26472 (62%)	-0.86	0.7	-0.06	-0.05
Basal	27 (30%)	16211/ 26472 (61%)	-0.9	0.84	-0.07	-0.07
HER2+	26 (30%)	15777/ 26472 (60%)	-0.91	0.79	-0.06	-0.06
LuminalA	13 (15%)	13047/ 26472 (49%)	-0.96	0.91	0	0
LuminalB	22 (25%)	15383/ 26472 (58%)	-0.88	0.77	-0.05	-0.05
* Corr = Correlation						

Supplementary Figure S4: Correlation values between DNA methylation and gene expression. Spearman correlation values are plotted as a histogram between methylation (Illumina's Infinium Assay) and gene expression (Affy HGU A133 Plus 2.0 chip). Summary statistics for all samples, as well as subtype classifications, are presented below.

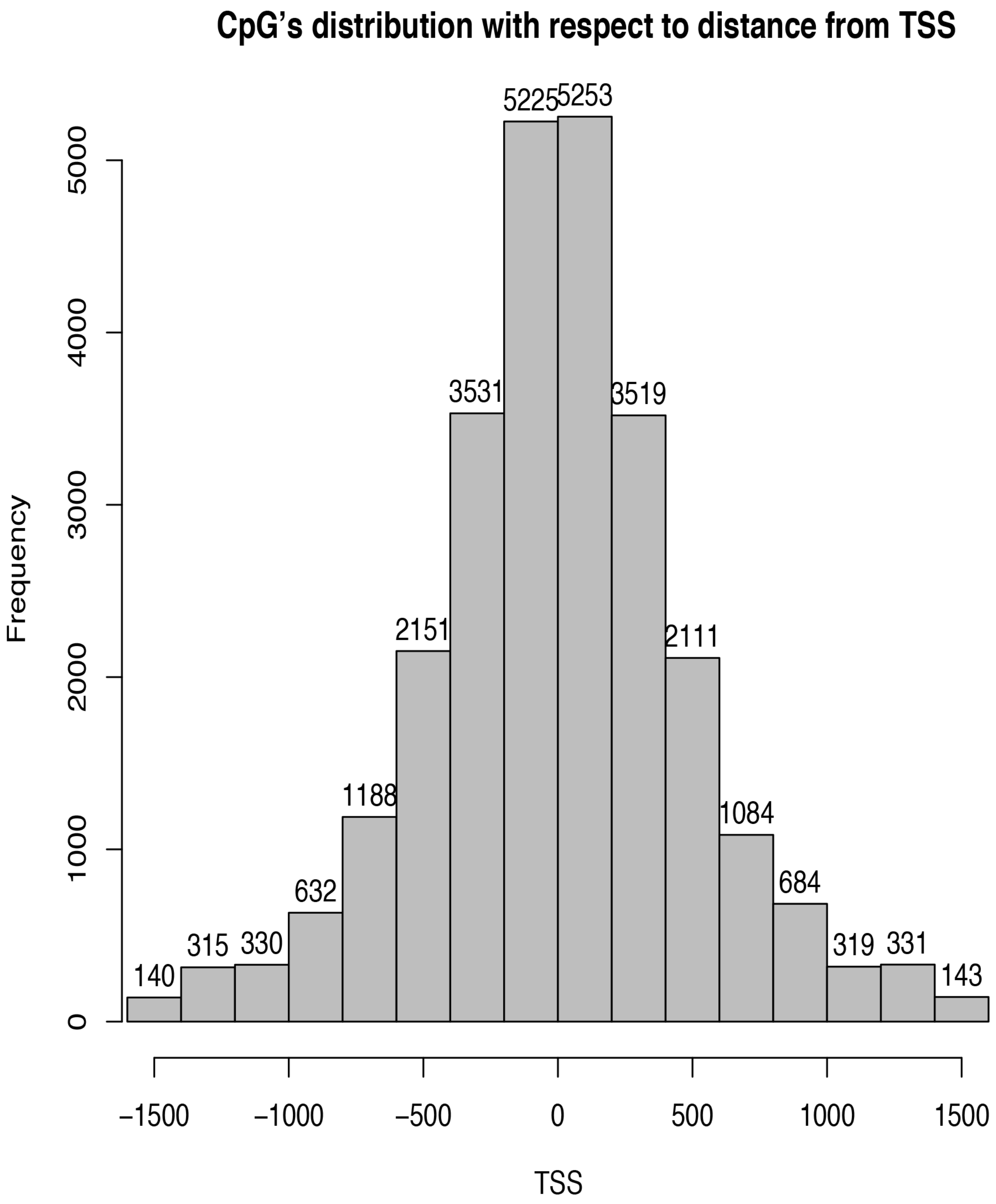

Supplementary Figure S5: Histogram of the global pattern of DNA methylation with respect to the distance from transcription start site (TSS) of Series 1. The X-axis shows the methylation data as a function of distance from the TSS, and the Y-axis shows the frequency of CpGs within that range.

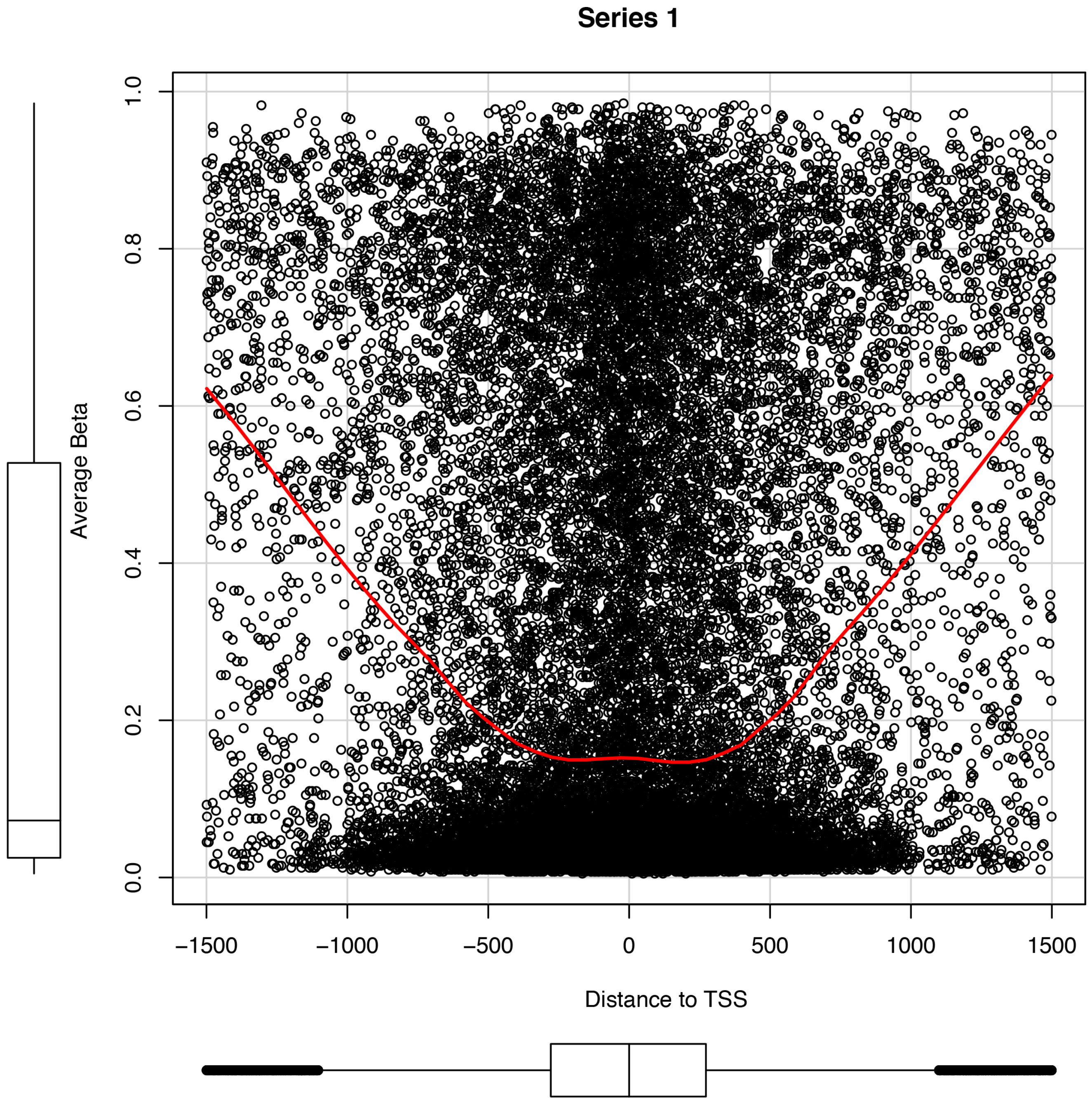

Supplementary Figure S6: Scatter plot of genome-wide associations between DNA methylation levels and the transcription start site (TSS). Each point in the scatter plot shows the mean methylation level for all normal samples from Series 1 data. The red line shows the smoothed curve. The data distribution according to mean methylation value is shown by boxplot (left), and according to the distance from the TSS is shown by boxplot (bottom).

Supplementary Table S1: The most significant differentially methylated CpGs across four breast cancer subtypes identify by Kruskal Wallis method and corresponding adjusted P-value using Bonferroni correction ("bonferroni"), Holm (1979) ("holm"), Hochberg (1988) ("hochberg"), Hommel (1988) ("hommel"), Benjamini & Hochberg (1995) ("BH" or its alias "fdr"), and Benjamini & Yekutieli (2001) ("BY"), respectively. Sheet Series1_KK corresponds to Series 1, and sheet Series2_KK corresponds to Series 2.

Supplementary Table S2: Correlation between between DNA methylation and gene expression for the common genes of Series 1 for all of the common samples (column All), basal samples (column Basal), HER2+ samples (column HER2+), luminal A samples (column luminal A), and luminal B samples (column luminal B).

Supplementary Table S3: Mapping tables: common genes identified between DNA methylation and gene expression of Series 1. (**A**) All possible CpGs corresponding to common genes between DNA methylation and gene expression annotation data. (**B**) If there were more than one CpG for the corresponding gene; most variant CpGs across four breast cancer subtypes the samples were identified.
